# P02-05 Our healthy community - development of a new model for health promotion and disease prevention in Danish municipalities

**DOI:** 10.1093/eurpub/ckac095.024

**Published:** 2022-08-29

**Authors:** Mette Aadahl, Paul Bloch, Charlotte Demant Klinker, Charlotta Holm Pisinger, Henrik Vardinghus-Nielsen, Thea Suldrup Jørgensen, Mette Kirstine Tørslev, Henrik Bøggild, Ulla Toft

**Affiliations:** 1 Center for Clinical Research and Prevention, Bispebjerg and Frederiksberg Hospital, Frederiksberg, Denmark; 2 Steno Diabetes Center Copenhagen, Health Promotion, Gentofte, Denmark; 3 Department of Health Science and Technology, Aalborg University, Aalborg, Denmark

**Keywords:** Health promotion, Co-creation, Supersetting, Prevention

## Abstract

**Background:**

The Danish health care system is facing major challenges as the prevalence of chronic diseases increases. There is a need for new approaches and strategies to prevent chronic disease and promote health and well-being among citizens. The aim is to describe the development of a new model for coordinated, integrated and evidence-based health promotion and disease prevention in Danish municipalities. The model builds on the supersetting approach, intersectoral collaboration and community engagement and applies a broad bio-psychosocial concept of health.

**Methods:**

Two Danish municipalities were included in the initial development and testing of the model from 2019 to 2021. This involved the following steps in each municipality: 1) Analyzing the health status, lifestyles and socio-economy at municipality level. 2) Mobilizing lead municipal administrators and politicians for intersectoral action including jointly defining thematic focus areas and target populations. 3) Mapping community-based stakeholders, physical environments and existing evidence to qualify relevant action 4) Mobilizing professional stakeholders from the public, private and civic sectors for co-creation of intervention ideas and joint action. 5) Co-creating and implementing interventions together with professional stakeholders and citizens.

**Results:**

The strategic model and results from the development process will be presented from one of the involved municipalities: The municipal administration chose physical activity and well-being among children and young people as their key focus area. Community-based stakeholders from non-profit organizations and public institutions, including sports clubs, leisure clubs, primary schools, and public departments jointly developed and implemented specific interventions. One specific intervention aimed to engage more children in local clubs. Coaches from three local sport clubs introduced 1st and 4th grade students at two schools to their sport (a course of eight times) during students' time in their local after-school club. Overall, the process fostered broad engagement of stakeholders from the public sector, the private sector, and civil society.

**Conclusion:**

The model developed in Our Healthy Community builds on contextual analyses, dialogues, workshops, and co-creation processes with a wide range of stakeholders to promote local relevance, integration and sustainability of developed actions and interventions. The model will be pilot tested in two other Danish municipalities (2022-2025)

